# Supplementary material for: “Back Health 24/7/365”—A Novel, Comprehensive “One Size Fits All” Workplace Health Promotion Intervention for Occupational Back Health among Hospital Employees
Source: Int J Environ Res Public Health. 2024 Jun 14;21(6):772. doi: 10.3390/ijerph21060772 (PMC11203411; doi:10.3390/ijerph21060772)
Supplement: Supplementary file 1 [file ijerph-21-00772-s001.zip › Supp File S1_Teaching contents.pdf]

Supplement File S1. Teaching contents of the WHP intervention “Back Health 24/7/365”:

- Practical session 1 – Manual material handling:
    - Basics
        - Raising awareness of external load & back strain/muscle activity
        - Experiencing lumbar spine muscle activity during ADLs
        - Movement within the spine vs. movement of the overall spine as a system
            - Spinal flexion vs. hip hinge => movement training with spine model
    - Weightlifting
        - How to lift and to put down a load?
            - Shorten the load arm => Message: “Carry the load as close as possible to the body.”
            - Pre-activation of spine extensor muscles => Message: “Do spinal bracing before you start to lift the weight and keep your spine static.”
            - Hip hinge => Message: “Do the dynamic lifting with the hamstrings and the glutes, not with the back.”
        - How to transfer weight from left to right?
            - Movement of the whole spine in the transversal plane vs. rotation and lateral flexion movements within the spine => Message: “Move the muscularly pre-stabilized spine as a whole and do not put any external stress on a twisted and bent spine.”
        - How to utilize weightlifting techniques for work situations and ADLs?
            - Message 1: “Due to environmental factors, it is not always possible to achieve the optimal spinal position. However, it is always possible to optimize the lifting technique as much as possible.”
            - Message 2: “Lifting incorrectly once will not cause lasting damage to the spine, so don't stress yourself out. If you get it right 90% of the time, you will benefit significantly.”

- Practical session 2 – Specifically counteracting sedentary behavior:
    - Stretching of muscles which tend to tighten due to sedentary behavior
        - Posture correcting
            - M. pectoralis major
            - M. iliopsoas & m. rectus femoris
            - M. sternocleidomastoideus
        - How to counteract tension-associated headaches
            - M. trapezius pars descendens
            - M. levator scapulae
            - M. scalene
        - How to counteract tension associated low back pain
            - M. glutaeus maximus
            - Mm. ischiocrurale
    - Strengthening of muscles which tend to get weak due to sedentary behavior
        - Dynamic strengthening of the back extensors (bent forward standing hyperextensions)
        - External rotators of the shoulder with resistance bands

        - Dynamic strengthening of the abdominal muscles: Ab-crunches, dynamic side-planks

- Practical session 3 – Core stability training
    - Core strengthening exercises
        - Standing row with resistance bands (bilateral, unilateral, alternating)
        - Planking (with variants)
        - Side-planking (static)
        - Glute bridge (with both legs, single-leg)
        - Supine hip flexor
        - Hip-abductor sidesteps with resistance band

  Great emphasis was placed on correct execution of all exercises in all practical units.

- Video session:
    - Guideline-based treatment of back pain
        - Primary-prevention
        - Guideline-based diagnostics (specific vs. non-specific back pain)
        - Guideline-based therapy
        - Active treatment
        - No sick note when treated according to guidelines for non-specific back pain!
    - Anatomy of the spine
        - Active musculoskeletal system
            - Spinal muscles
            - Abdominal muscles
        - Passive musculoskeletal system
            - Bones
            - Intervertebral discs
        - Nervous system
            - Spinal cord
            - Spinal nerves
    - Mental health and back pain
        - Psychological risk factors for the chronification of back pain
        - Chronic distress
        - Pain coping strategies
    - Nutrition and back pain
        - How overweight shifts the body’s center of gravity and how this affects spinal kinematic and muscles
        - Practical advice of how to keep a healthy weight
