# Supplementary figures and images for: “Back Health 24/7/365”—A Novel, Comprehensive “One Size Fits All” Workplace Health Promotion Intervention for Occupational Back Health among Hospital Employees
Source: Int J Environ Res Public Health. 2024 Jun 14;21(6):772. doi: 10.3390/ijerph21060772 (PMC11203411; doi:10.3390/ijerph21060772)

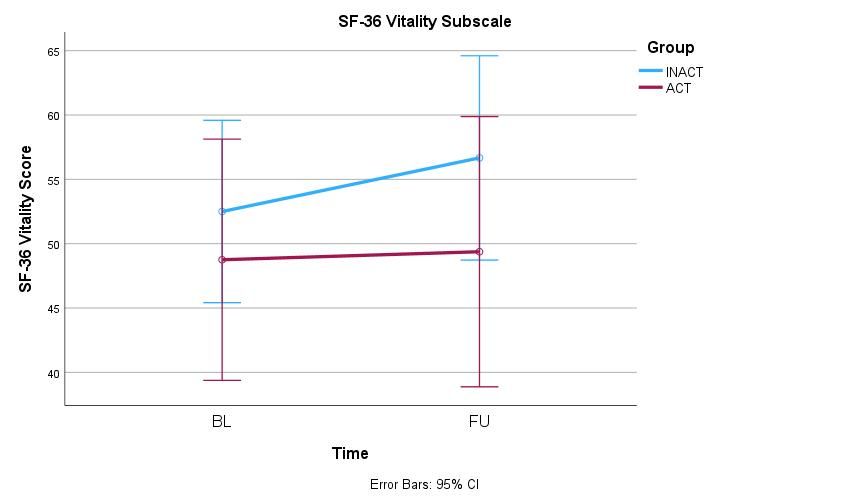

Supplement: Supplementary file 1 [file ijerph-21-00772-s001.zip › Supp_Figure S10_SF-36_Vitality_300dpi.jpg]

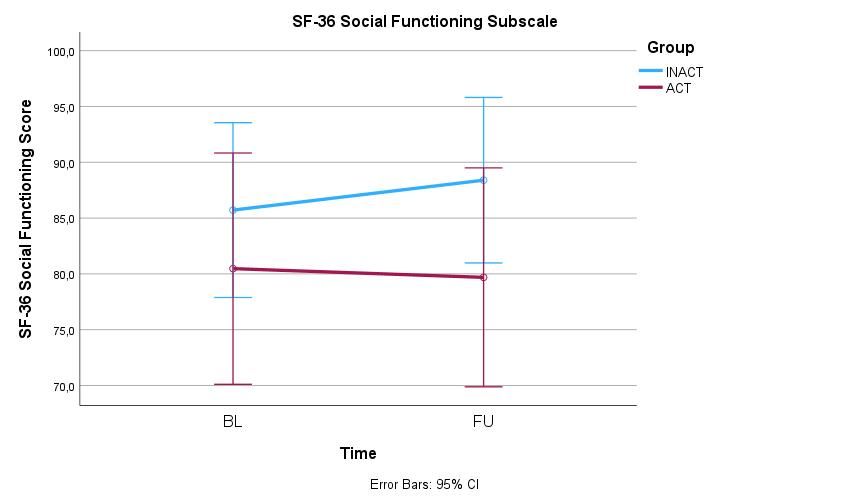

Supplement: Supplementary file 1 [file ijerph-21-00772-s001.zip › Supp_Figure S11_SF-36_Social Functioning_300dpi.jpg]

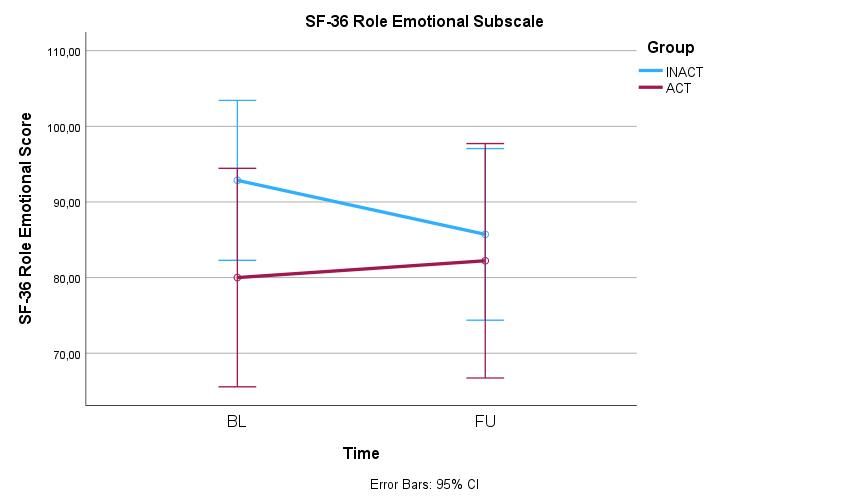

Supplement: Supplementary file 1 [file ijerph-21-00772-s001.zip › Supp_Figure S12_SF-36_Role Emotional_300dpi.jpg]

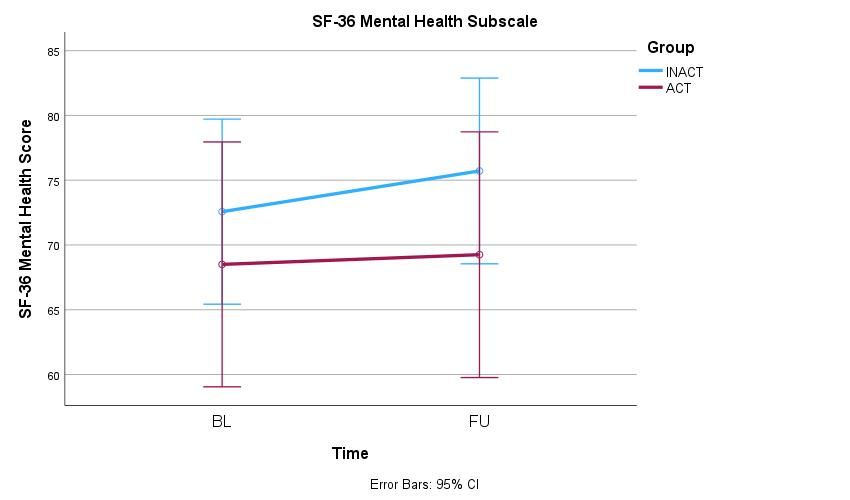

Supplement: Supplementary file 1 [file ijerph-21-00772-s001.zip › Supp_Figure S13_SF-36_Mental Health_300dpi.jpg]

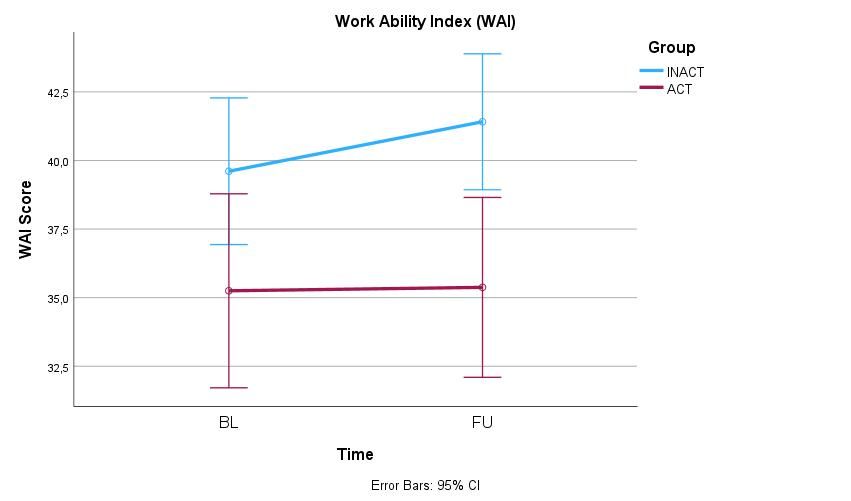

Supplement: Supplementary file 1 [file ijerph-21-00772-s001.zip › Supp_Figure S1_WAI_300dpi.jpg]

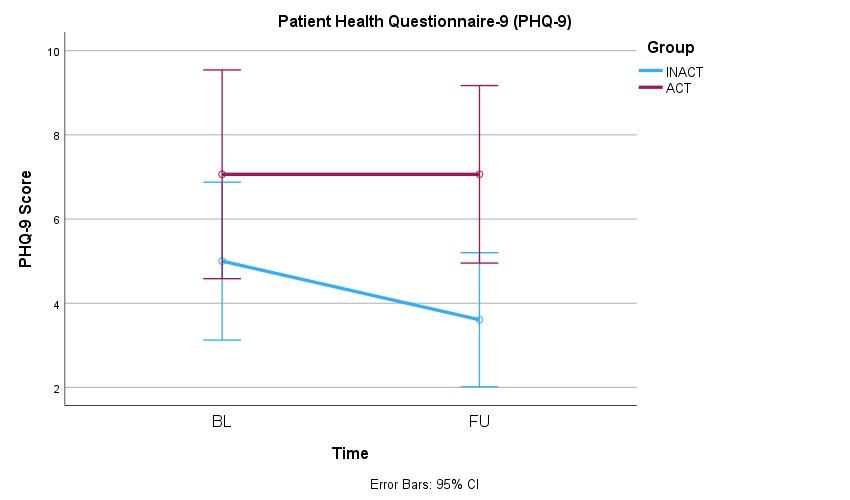

Supplement: Supplementary file 1 [file ijerph-21-00772-s001.zip › Supp_Figure S2_PHQ-9_300dpi.jpg]

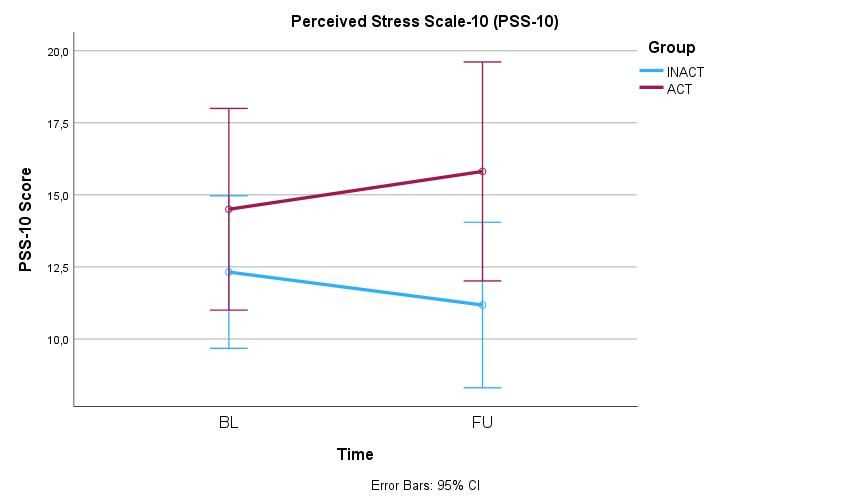

Supplement: Supplementary file 1 [file ijerph-21-00772-s001.zip › Supp_Figure S3_PSS-10_300dpi.jpg]

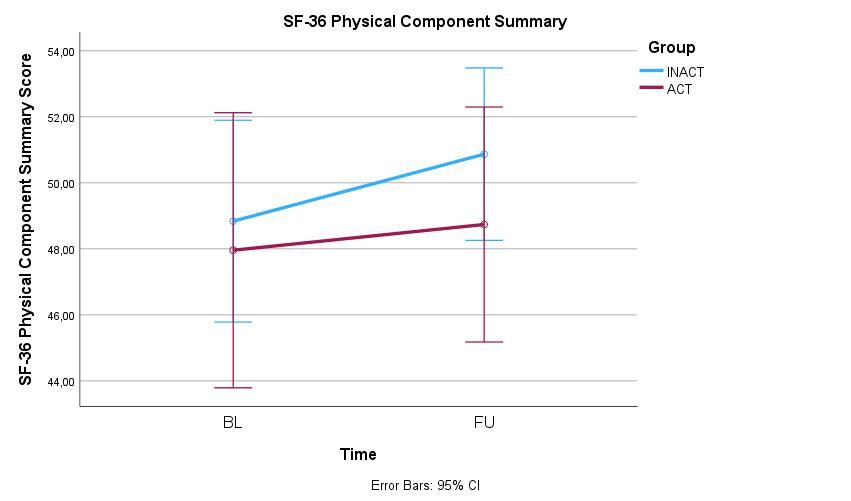

Supplement: Supplementary file 1 [file ijerph-21-00772-s001.zip › Supp_Figure S4_SF-36_Physical Component Summary_300dpi.jpg]

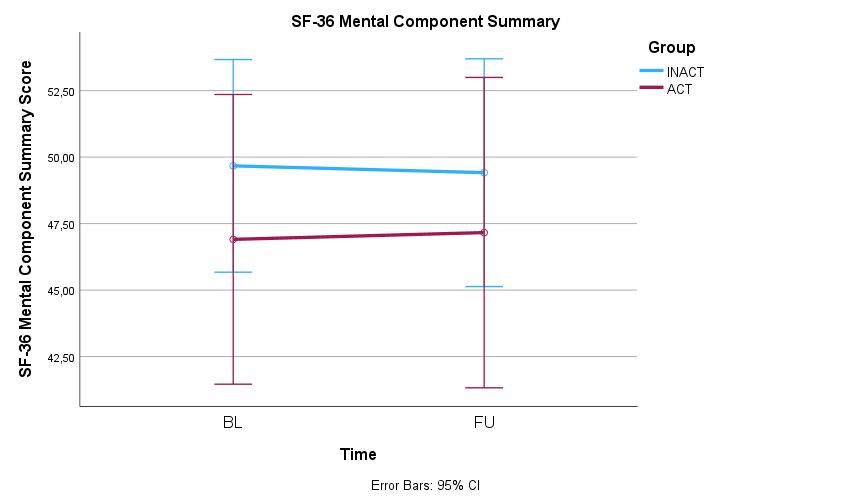

Supplement: Supplementary file 1 [file ijerph-21-00772-s001.zip › Supp_Figure S5_Mental Component Summary_300dpi.jpg]

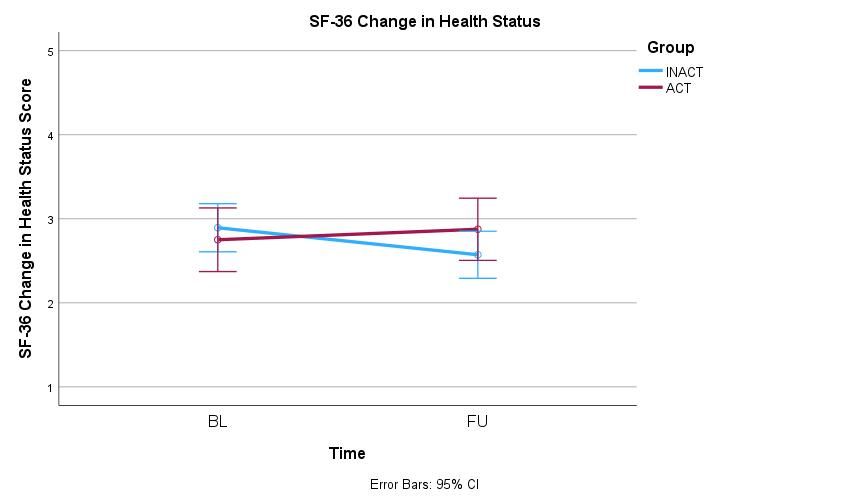

Supplement: Supplementary file 1 [file ijerph-21-00772-s001.zip › Supp_Figure S6_SF-36_Change in Health Status_300dpi.jpg]

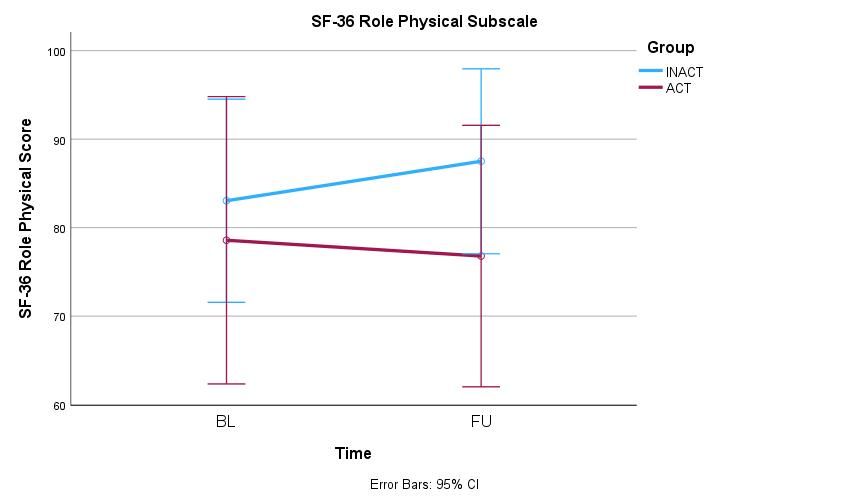

Supplement: Supplementary file 1 [file ijerph-21-00772-s001.zip › Supp_Figure S7_SF-36_Role Physical_300dpi.jpg]

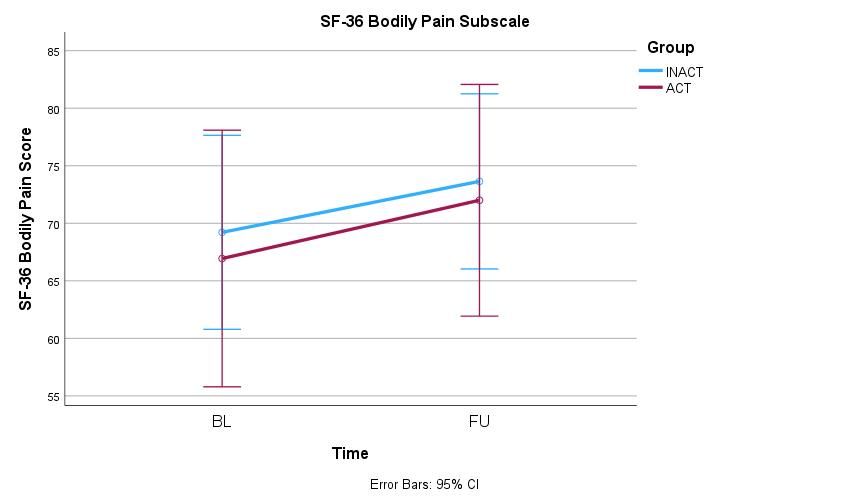

Supplement: Supplementary file 1 [file ijerph-21-00772-s001.zip › Supp_Figure S8_SF-36_Bodily Pain_300dpi.jpg]

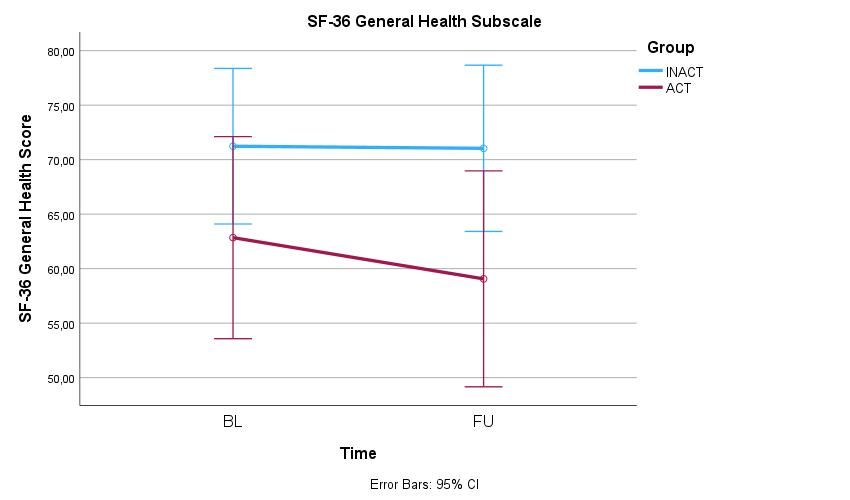

Supplement: Supplementary file 1 [file ijerph-21-00772-s001.zip › Supp_Figure S9_SF-36_General Health_300dpi.jpg]
